# 10-Valence-Electron C≡O and the 14-VE C≡Pt: Two Triple-Bonded Isoelectronic Families Differing by a *d*δ^4^ Ring

**DOI:** 10.1021/acs.inorgchem.3c02889

**Published:** 2023-12-05

**Authors:** Michiko Atsumi, Pekka Pyykkö

**Affiliations:** † Hylleraas Centre for Quantum Molecular Sciences, Department of Chemistry, University of Oslo, Norway. Postboks 1033, Oslo 0315, Norway; ‡ Department of Chemistry, Faculty of Science, University of Helsinki, P.O.Box 55 (A. I. Virtasen aukio 1), Helsinki 00014, Finland

## Abstract

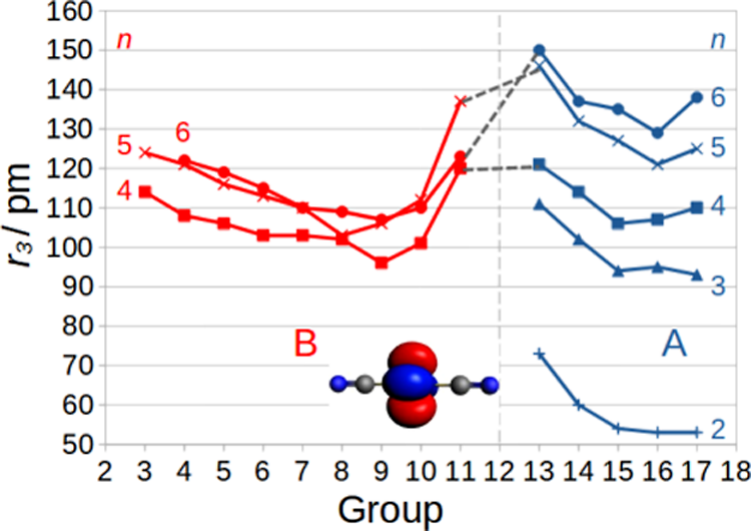

10-VE A≡A′ Diatomics, such as N≡N, C≡O, etc., have a strong σ^2^π^4^ triple bond plus a lone pair at each end. In our studies on 14-VE A≡B systems, such as C≡Pt, we find a similar bonding system plus a (5*d*δ)^4^ ring. Here, the A atom belongs to groups 13–17 and the B atom to groups 7–11. Also the BB′ combinations, triatomics, such as PtCO or DsCO or uranyl, and longer chains, such as AuCN and [NC-Au-CN]^−^, are discussed. The δ ring directly contributes to nuclear quadrupole coupling constants, and DFT calculations using the BH and H or mPW1K functionals reproduce the experimental trends of the NQCC.

## Abstract 2

Systems like C≡Pt have C≡O-like σ_2_π_4_ bonds plus a dδ_4_ ring, formally in the core but energetically and sizewise in the valence range. It dominates the nuclear quadrupole coupling of the delement. A polyatomic δ_4_ example is the important [Au(CN)_2_]^−^.

## 1 Introduction

While developing a set of additive covalent triple-bond radii, *r*_3_,^1,2^ the same covalent radii *r*(*A*) were used in fitting the main-group A≡A′ and the A≡B bond lengths, where B is a transition metal. The primary bond lengths came from experimental or computed closed-shell systems with main-group elements and with transition metals as long as the molecular-orbital inspection and the actual bond length fitted the picture. The same covalent radii *r*(*A*) were used in fitting the main-group A≡A′ and the A≡B bond lengths for both single, double, and triple bonds, *r*_1_, *r*_2_, and *r*_3_, respectively.

In our ongoing calculations, we kept finding two different sets of diatomic species. One was the well-known 10VE family of diatomic systems, such as N_2_, CO, CN^–^, NO^+^, etc. These are known to have a nominal σ^2^π^4^ triple bond combined with a lone electron pair at each end. In all, we then have 10 valence electrons (10VE)^3^ (it should be noticed that, as an alternative to this triple-bond picture, in older literature, a C=O double bond was thought to exist, and also other resonance hybrids have been mentioned, see Pauling^4^ or Long and Walsh^5^).

The other families, such as CPt, *X*^1^Σ had four more electrons, always occupying a δ^4^ ring, typically the penultimate, HOMO – 1. With this, we mean the occupied (d_*xy*_)^2^(d_*x*^2^–*y*^2^_)^2^ combination, in an alternative form , or (*d*δ)^4^. The novelty is that we have an occupied orbital which is formally a nonbonding core orbital but has the size and orbital energy of a typical valence orbital. That observation is not new, and some previous examples are shown in Table 1. We are, however, not aware of any previous articles concentrating on this feature.

**Table 1:** Examples on δ^4^ Systems

year	species	ref
1998	CAu^+^	Barysz,^8^ Pyykkö^9^
1999	NIr	Ram^10^
2003	SiPt	Barysz^11^
2004	CPt	Patzschke^12^
2015	SiNi	Schoendorff^13^
1985	[XAuX]^−^	Bowmaker^14^
1985	[NC-Au-CN]^−^	Bowmaker^14^

Starting from these 10-VE or 14-VE families of species, we made some further contacts:

1 It also is possible to have the δ^4^ ring without any triple bonds to the metal.
2 In multiple bonds between two B atoms, bond orders beyond 3 can be reached, see Cotton et al.^6^
3 One way to “see” the δ^4^ electrons is to measure nuclear quadrupole coupling constants. While many DFT functionals have problems in reproducing the electric field gradient (EFG), *q*, at transition-metals,^7^ we have now found two functionals that work. We also report some results for species, not involving the δ^4^.

## 2 Methods

We use in the present calculations DFT and ZORA at either the scalar relativistic (SR) or spin–orbit (SO) level. This will provide reasonable accuracy and easy interpretation of chemical bonding questions.

The calculations were carried out by using the ADF software package^15^ with the Perdew–Burke–Ernzerhof (PBE) functional during the structural calculations. The triple-ζ basis sets with two polarization functions (TZ2P) were used for all elements, treated at the nonfrozen core level.

We have added into Tables 2, 3, 4, 5, and 6 the results of some earlier calculations and a comparison with the sum^a^ of our triple-bond covalent radii *r*_3_.^1,2^

**Table 2:** Calculated and Experimental Bond Lengths, *R*_e_, for AB Diatomics^a^

	*R*_e_/pm				
AB	PW	exp	calc	*r*_3_	δ
BRh^d^	170.36^b^	169.2	168.5^16^	179	H
BPt^–^	181^b^		179.90^1^	183	H-1
	180.9^c^				
BAu	192.1^b^		192.27^1^	196	H-2
	191.6^c^		190.6^17^		H-1
CNi	160.5^b^	162.73^18^	159.60^1^	161	H-1
CRu^d^	160.9^b^	160.5485(2)^19^	160.1^19^	163	H
			160.42^20^		
CPd	172.2^b^	172.2	171.6^1^	172	H-1
	172.3^c^				
CPt	168.3^c^	167.7^21^	172.5^12^	170	H-1
	167.8^b^		167.45^1^		H-1
CAu^+^	178.9^b^		176.6^8,9^	183	H-2
	178.8^c^				
CDs	172.2^b^		172.7^12^	172	
	172.6^c^				
NIr	160.8^c^	160.68281(35)^10^	160.9^10^	161	H-1
SiNi	201.2^b^	203.165(24)^22^	208.6^13^	203	H
SiPt	208.2^b^	206.29(2)	210^11^	212	H-1
	208.7^c^				
SiAu^+^	219.3^b^		218.8^8^	225	H-2
	219.4^c^				
PIr	198.3^b^	199.28^e^^,^^23^		201	H
	199.9^c^				
TlAu	269^b^			273	H-1
	266.6^c^				
BiP	229.5^b^	229.61520(80)^24^		229	
	230.2^c^				
BiAu	258.6^b^			258	H-1
	263^c^			258	H-2,H-3

a δ gives the energetic placement of the *d*δ^4^ MO with respect to the HOMO (H). “PW”: Present work. *r*_3_: From triple-bond covalent radii.^1,2^

b SR.

c SO.

d 12VE species.

e *R*_0_.

**Table 3:** Calculated and Experimental Bond Lengths, *R*_e_ for BB′ Diatomics^a^

	*R*_e_/pm				
BB′	PW	exp	calc	*r*_3_	δ
Cr_2_	159.5^b^	167.88	156.2^28^	206	H
CrU	185.4^b^		188.3^27^	221	H
	187.6^c^				
MoU	202.8^b^		202.1^27^	231	H
	205.9^c^				
WU	208.5^b^		208.0^27^	233	H
	217.5^c^				
ThU	248.2^b^			254	H
	279.3^c^				H
ThPt	237.3^b^	254^29^	250^11^	246	H
	237.7^c^				
ThAu^+^	271.6^b^		263^1^	259	H-1
	262.4^c^				

a δ gives the energetic placement of the *d*δ^4^ MO with respect to the HOMO (H). PW: Present work. *r*_3_: From triple-bond covalent radii.

b SR.

c SO.

**Table 4:** Calculated and Experimental Bond Lengths, *R*_e_, for Linear Polyatomic Species^a^

	*R*_e_/pm					
species	bond	PW	exp	calc	*r*_3_	δ
AuCN^c^	Au–C	190.6	191.51^45^		183	H-2
	C–N	116.9	115.56^45^		114	
[NCAuCN]^−1^^b^	Au–C	198.8	198.4^d^	198.8^43^	183	H-2
				199^46^		
[HAuCN]^−1^	Au–C	207.4^b^		202.5^47^	183	H-2
		202.1^c^				
[FAuF]^−^	Au–F	199.9^b^		196.3^43^	176	H-2
		199.3^c^				
[ClAuCl]^−^	Au–Cl	230.2^b^	228	228.3^43^	216	H-2
		229.6^c^		228.3^48^		
[BrAuBr]^−^	Au–Br	242.2^b^	240	239.4^43^	233	H-2
		241.5^c^		240.8^48^		
[IAuI]^−^	Au–I	259.6^b^	253	256.1^43^	248	H-2
		259.2^c^		256.9^48^		
[AtAuAt]^−^	Au–At	268.6^b^		263.9^43^	261	H-3
		270.2^c^				
PtCO	Pt–C	176.1^b^	176.046^49^	177.6^30^	170	H
PtCPt	Pt–C	173.8^c^		173.5^30^	170	H-2
[AuCAu]^2+^	Au–C	182.6^b^		173.5^30^	183	H-2
		182.4^c^				
NUIr	N–U	173.9^b^		172.2^32^	172	H-1
		179.8^c^				
	U–Ir	217^b^		218.4^32^	225	H-1
		222.5^c^				
OUPt^2+^	O–U	172.7^b^		171.4^50^	171	H-1
		174.3^c^				
	U–Pt	228.6^b^		221.4^50^	228	
		229^c^				

a The [XAuX]^−^ experimental values for X = Cl–I are taken from.^44^ In the last column, the δ gives the energetic placement of the *d*δ^4^ MO with respect to the HOMO (H). Slight variations of the order may occur as a function of the method. PW: Present work. *r*_3_: From triple-bond covalent radii. “Calc.” refers to earlier calculations.

b SR.

c SO.

d In Nd[Au(CN)_2_]_3_·3H_2_O.^51^

**Table 5:** Vibrational Frequencies, ω_e_ (cm^–1^), for Diatomics

	ω_e_/pm		
species	PW	exp	calc
BRh^c^	939^b^		
BPt^–^	844^a^		
	842^b^		
BAu	658^a^		
		704^17^,^d^	710^17^
BAu	663^b^		
CNi	959	875.155^18^	
CRu^c^	1156^b^	1100^19^	
CPd	885^a^		
	881^b^		
CPt	1059^b^	1051.13(2)^21^	1096^12^
CAu^+^	827^a^		873^8^
	826^b^		
CDs	1115^b^		1147^12^
NIr	1166^b^	1126.1757(28)^10^	1161^10^
		1195(38)^54^^,^^e^	
SiNi	499^a^	467.43^22^	458^13^
	497^b^		
SiPt	537^a^	549.0(3)	531^11^
	527^b^		
SiAu^+^	427.2^a^		459^8^
	426.6^b^		
PIr	624^a^	570^23^	
	591^b^		
TlAu	135^a^		
	139^b^		
BiAu	168^a^	157.7^55^	
ThPt	248^a^	221^29^	211^11^
	245^b^		
ThAu^+^	143^a^		188^11^

a SR.

b SO.

c 12VE species.

d *r*_0_.

e In noble-gas matrices.

**Table 6:** Vibrational Frequencies for Polyatomic Species^b^^,^^c^

		ω_e_/cm^–1^		
species	mode	PW	exp	calc
AuCN	ν_1_(σ)	2161		2276,^45^ 2181^56^
	ν_2_(π)	335	320^45^	313″, 285^56^
	ν_3_(σ)	474	480^45^	476″, 472^56^
[Au(CN)_2_^–^]	C–Nσ_u_	2130	2158^a^,^51^	
	C–N σ_g_	2147	2166^51^	
	Au–C σ_u_	403	474, 491^51^	
	Au–C π_u_	420		
	Au–C σ_g_	427		
	π_g_	290		
	π_u_	80		
AuCl_2_^–^	σ_g_	300.6	329^57^	310^43^
	σ_u_	320.9	350^57^	330″
	π_u_	110.5	116^57^	103″
AuBr_2_^–^	σ_g_	193	209^57^	194″
	σ_u_	238	254^57^	238″
	π_u_	74.6	77^57^	69″
AuI_2_^–^	σ_g_	142	158^57^	143″
	σ_u_	200	210^57^	194″
	π_u_	55.8	63^57^	52″

a In solid Nd[Au(CN)_2_]_3_·3H_2_O.

b SR.

c 12VE species.

## 3 Results

### 3.1 Diatomics

**Figure 1:**
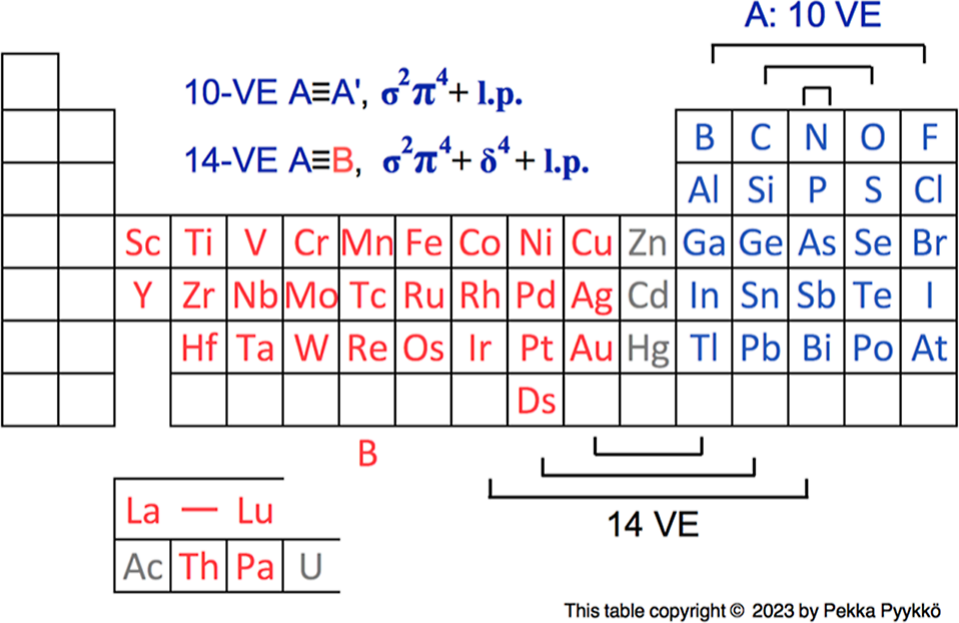
Schematic placement of the main-group elements (A) and transition elements (B). The 10VE A≡A′ and 14VE A≡B combinations are indicated. l.p. = lone pairs.

Table 2 gives the bond lengths of the A≡B diatomics, specified in Figure 1, mostly for 14VE species.

These AB *R*_e_ values in Table 2 are close to the sum of triple-bond covalent radii. To the contrary, the BB′ ones in Table 3 can be much shorter, reflecting a bond order higher than three. Quadruple bonding in ground or excited states of certain AB diatomics^20,25^ and BB systems^6^ has also been discussed.

Liu et al.^26^ consider the donation and its direction in certain multiply bonded AA′ diatomics. Cheung et al.^16^ also discuss 4-fold bonding in RhB. The MU species (M = Cr–W) of Ruipérez et al.^27^ have the δ ring as the HOMO, see Table 3. Note that at the scalar relativistic level, these are 12-VE species with a (1π)^4^(1σ)^2^(2σ)^2^(1δ)^4^*X*^1^Σ ground state, see ref (27)Figure 1.

### 3.2 Polyatomic Chains

If group 11 (Cu, Ag, Au, and Rg) doubles as a halogen and group 10 (Ni, Pd, Pt, and Ds) as a chalcogen, it is easy to take the step from OCO to PtCO or DsCO.^12^ With metals at both ends, we have [Au=C=Au]^2+^, or the analogues CPt, CPt_2_, and CPt_3_^2–^ to CO, CO_2_, and CO_3_^2–^, respectively.^30^

Of the species in Table 4, the triatomic PtCPt and [AuCAu]^2+^ have two δ^4^ rings, both a *g* and a *u* one, separated from each other by the carbon atom.

Similarly, the uranyl isoelectronic series^31^ would yield NUN and the NUIr of Gagliardi and Pyykkö.^32^ The NUO^+^ predicted by Pyykkö et al.^31^ was later made by Heinemann and Schwarz^33^ in the gas phase and by Zhou and Andrews^34^ in Ne matrices. The OUIr^+^ was prepared by Santos et al.^35^ For reviews on uranyl analog complexes, see Wei et al.,^36^ or Maria and Marçalo.^37^ A closer analysis of U≡A multiple bonding was given by Motta and Autschbach.^38^

With 5d metals at both ends, linear species like PtThIr^–^ were found by Hrobárik et al.^39^ This species had a δ^4^ ring at the Pt end, and some evidence for Th–Ir δ bonding at the Ir end. Two of the σ bonds were characterized as “a sausage inside a tube”.

The isoelectronic series of [ClAuCl]^−^ can be continued at least to the high-pressure compound Li_5_AuP_2_.^40^ The energetic location of the δ^4^ ring in the [XAuX]^−^ series, X = F–At, is discussed in the last column of Table 4.

Pauling^41^ considered a δ bond in Re_2_Cl_8_^2–^, an assigned quadruple-bond case. For further examples, see the book.^6^ He also found two triple U≡O bonds in uranyl. Possible members of the uranyl isoelectronic series down to [CUC]^2–^ have been discussed.^31^

**Figure 2:**
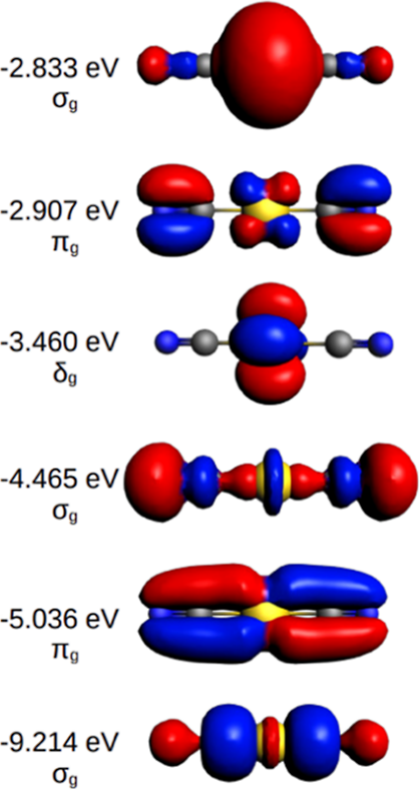
Highest occupied MO/s of a free [NC-Au-CN]^−^ anion. The δ^4^ ring is the HOMO – 2 one at −3.460 eV.

Most of the World’s gold production^42^ is based on the [NC-Au-CN]^−^ ion, which has a beautiful δ ring, as seen from Figure 2. This δ energy level occurred as H-2 also in earlier calculations.^43^

### 3.3 Other Properties

#### 3.3.1 Relation of Bond Lengths and the Triple-Bond Covalent Radii

Both A≡A′ and A≡B triple bonds with various principal quantum numbers, *n*, were used to originally fit the additive *r*_3_. As seen from Tables 2 and 3, the present *R* mostly agree with
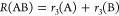
1for triple bonds. The higher bond-orders in Table 3 may be substantially shorter.

**Figure 3:**
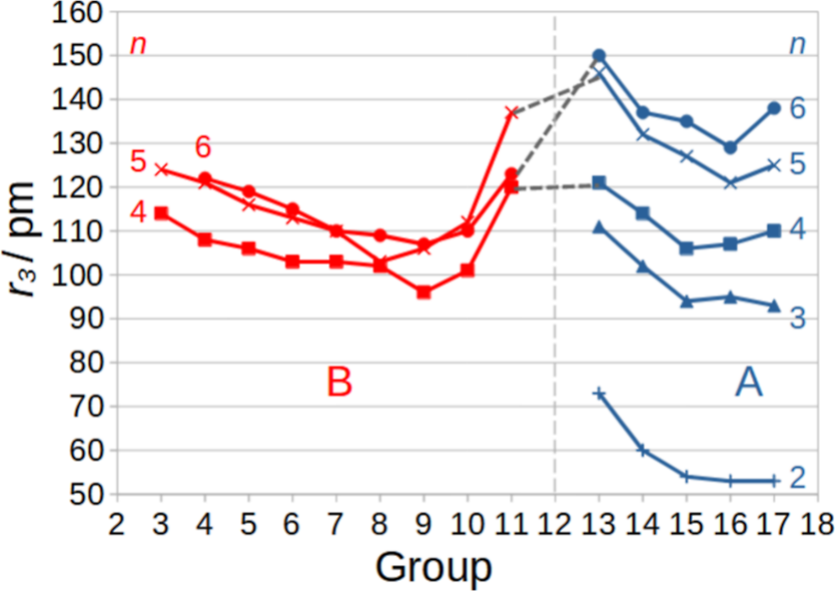
Additive triple-bond covalent radii, *r*_3_ (in pm), for transition-metal atoms (B) and main-group elements, (A) belonging to the groups 3–11 and 13–17, respectively. *n* is the number of the row.

Clear trends as a function of the group and the row are seen in Figure 3. For the shortness of the *n* = 2 radii, see Wang et al.^52^ Note for the *n*-dependence of the main-group elements A the clear trend 2 ≪ 3 < 4 < 5 < 6, while the transition metals, B, in the groups 9–10 rather have 6 < 5 ≈ 4. The sixth-row elements near gold have a local maximum of the relativistic bond-length contraction.^53^

Vibrational frequencies are another direct connection to experiments. As examples, we can take Table 5 on diatomics or Table 6 on polyatomic chains.

Dissociation energies and predissociation of species like CuB, AuB, or AlB are discussed by Merriles and Morse.^58^ The δ MO is quoted in their Supporting Information.

Nuclear quadrupole coupling constants, NQCCs, give a direct access to the electric field gradient, *q* at the nuclei involved. The four electrons in the δ^4^ ring make a major contribution. The RuC has been mentioned as an example by Wang et al.,^19^ who estimate that +1350 MHz of the experimental *B*_0_ of +433 MHz of ^101^RuC come from the δ^4^ ring. Without this contribution, even the sign would be wrong. Gusmão et al.^59^ obtained similar *q* values for RuC.^b^

For nuclear quadrupole coupling constants, BHandH and mPW1K functionals were applied. The BHandH gave earlier good *q* for HCl and CuCl,^60^ CdMe_2_,^61^ and Cd(SMe)_2_.^62^

In early multiple-scattering Xα calculations, Bowmaker et al.^14^ gave an orbital-based analysis of their *q* and found for [Au(CN)_2_^–^] that about +15.805 of the total *q* of −9.650 au arise from the H-4 δ^4^ electrons. They emphasized in their scalar-relativistic discussion of the AuX_2_^–^ species the *q* contributions from the Au 6p_*z*_ orbital.

**Figure 4:**
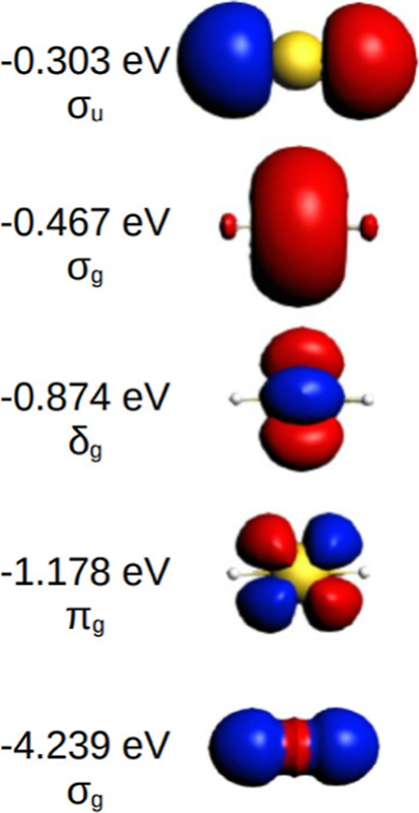
Highest occupied orbitals of the AuH_2_^–^ anion. The result resembles Figure 6 of Liu et al.^64^

In the linear HAuH^–^ both the Au 5dπ and 5dδ would be “inert” and only the Au 6s and 5dσ participate in bonding, as seen from Figure 4. The AuH_2_^–^ anion has been seen in matrix spectroscopy^63^ and photoelectron spectroscopy.^64^

In actual fact, one must emphasize that the XAuX^–^ calculations show some unusually strong dependence on the chosen functional.^57^ Moreover, the Mössbauer measurements are performed on solid samples with so far unknown matrix effects.

**Figure 5:**
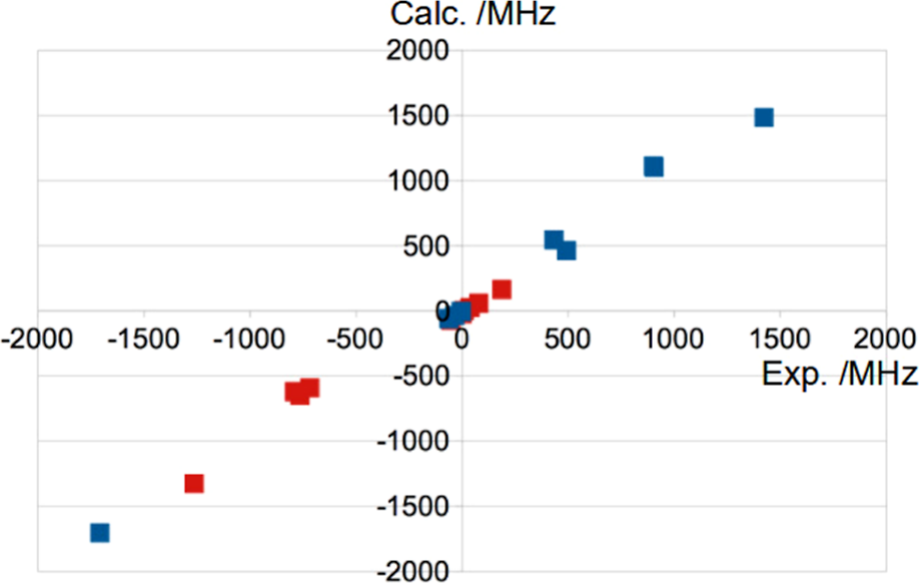
Comparison between calculated (PW) and experimental *B*. The data are taken from Tables 7 and 8. The red dots refer to *B*(Au) and the blue dots refer to other nuclei.

The gas-phase measurements included in Tables 7 and 8 have a chance to provide cleaner comparisons. The gas-phase microwave quadrupole coupling constants of Okabayashi et al.^65^ for triatomic AuCN were in complete disagreement with our earlier calculations, while their structural and vibrational AuCN parameters are in adequate agreement both with our more approximate work, and the latest and best studies.^56,66^ Then, we found that the alternative functionals BHandH and mPW1K gave a semiquantitative agreement between the experimental and calculated *B*, as seen from Figure 5.

**Table 7:** Calculated (SO) and Experimental NQCC, *B*_e_ = *e*^2^*qQ*/*h* (MHz), at the SO Level, Assuming the *Q* Values in Table 8

		*B*_e_/MHz		
species	Nucl	PW	exp	calc
BRh	^11^B	–1.29^b^		
BPt^–^	^11^B	–3.47		
BAu	^11^B	–4.41		
	^197^Au	260.0		
CNi	^61^Ni	89.2		
CRu^c^	^101^Ru	544.7	433.19(8)^19^	476.24^c^^,^^19^
CPd	^105^Pd	370.5		
CAu^+^^a^	^197^Au	322.9		
NIr	^14^N	–4.69		
	^103^Ir	1951.7	1721^c^^,^^71^	
SiNi	^61^Ni	78.0		
SiAu^+^	^197^Au	219.5		
PIr	^103^Ir	1484.2	1424^c^^,^^71^	
BiN	^209^Bi	1101.3	905.066(88)^24^	
	^14^N	–2.91	–2.468(13)^24^	
BiP	^209^Bi	1110.0	903.031^24^	
TlAu	^197^Au	93.5		
BiAu	^209^Bi	–237.4		
	^197^Au	151.3		
ThPt	^229^Th	–3759.1		
CrU	^53^Cr	–1.59		
	^235^U	1547.3		
MoU	^95^Mo	6.87		
	^235^U	291.4		
WU	^235^U	6823.2		
AuH	^197^Au	164.3	187.116(99)^72^	
AuD	^197^Au	164.3	188.119(33)^72^	
AuF	^197^Au	–72.6	–52.2344(67)^73^	
AuCl	^197^Au	2.61	9.63312(13)^74^	
	^35^Cl	–60.1	–61.99694(81)^74^	
AuBr	^197^Au	25.9	37.2669(14)^74^	
	^79^Br	463.8	492.3271(12)^74^	
AuI	^197^Au	58.5	78.273(11)^75^	
	^127^I	–1703.9	–1707.881(25)^75^	

a Assumed Q/mb from Pyykkö,^76^ unless otherwise stated: ^11^B 40.59(10), ^14^N 20.44(3), ^53^Cr 150(50), ^61^Ni 162 mb, ^101^Ru 457(23), ^100^Rh 153, ^105^Pd 660(11), ^193^Ir 751(9), ^197^Au 547(16). ^209^Bi 422(3)^77^ and references there. ^229^Th 3110(60), ^235^U 4936(6).

b 12VE species.

c At *v* = 0.

**Table 8:** Calculated (SO) and Experimental NQCC, *B*_e_ = *e*^2^*qQ*/*h* (MHz), at the SO Level for Polyatomic Species, Assuming the *Q* Values^a^

		*B*_e_/MHz		
species	Nucl	PW	exp	calc
AuCN	^197^Au	–23.2	–0.7246(46)^65^	
	^14^N	–5.01	–4.1522(17)^65^	
AuCl_2_^–^	^197^Au	–648.3	(−)765^b^, (−)802	–862.5^14^
				–721^14^
			(−)785^76^	
	^35^Cl	–35.6	(−)35.2^14,79^	
AuBr_2_^–^	^197^Au	–620.3	(−)790(50), (−)794^78^	–692^14^
	^79^Br	248.9		202^14^
AuI_2_^–^	^197^Au	–590.4	(−)718, (−)719^78^	–709^14^
Au(CN)_2_^–^	^197^Au	–1325.9	(−)1263	–1118^14^
			(−)1206^78^	
AuAt_2_^–^	^197^Au	–488.2		
AuD_2_^–^	^197^Au	–1347.3		
HAuCN^–^	^197^Au	–1348		
	^14^N	–4.80		
NUIr	^14^N	–1.30		
	^235^U	–4308.6		
	^193^Ir	1139.1		
OUPt^2+^	^17^O	0.84		
	^235^U	–1199.1		

a Assumed *Q*/mb from Pyykkö,^78^ unless otherwise stated: ^11^B 40.59(10), ^14^N 20.44(3), ^53^Cr 150(50), ^61^Ni 162 mb, ^101^Ru 457(23), ^100^Rh 153, ^105^Pd 660, ^193^Ir 751(9), ^197^Au 547(16). ^209^Bi 422(3)^77^ and references there. ^229^Th 3110(60), ^235^U 4936(6). The Au NQR frequencies of Bowmaker^14^ have been scaled by a Q-ratio of (547 mb/590 mb) = 0.927.

b See^80^.

As one point of reference, the neutral Au atom in a 5d^9^6s^22^*D*_5/2_ state has a *B* of −1049.781(11) MHz.^67^ In other words, one *d*^–1^ hole gives a GHz. Many of the red dots in Figure 5 are far smaller than that.

Concerning the spin–orbit effects and other relativistic effects on *q*, at the level of multiplicative correction factors, we refer to Pyykkö and Seth.^68^

The MNC ← MCN conversion has been calculated by Jana et al.^69^ Hill et al.^70^ quote a *B*_e_ of 2.80 MHz for an unspecified nucleus. They discuss the vibrational dynamics in detail.

Wang^46^ observed photoelectron spectra in [Au(CN)_2_]^−^(*g*) and found in calculations significant covalent character in the Au–C bond.

### 3.4 Recent Further Examples on δ^4^ Rings

Kalita et al.^82^ suggest RhSc as a sextuply bonded diatomic with a Rh → Sc δ HOMO. Likewise, Tzeli and Karapetsas^20^ find *X*^1^Σ^+^ ground states for RuC, RhB, and PdBe, all three having nonbonding δ^4^ HOMOs. For systems of the [MUM] type with M = Rh, Ir, see Shen et al.^83^ and references there.

## 4 Conclusions

The δ^4^ ring lies energetically and radially in the valence range of the 5d elements discussed despite being formally a filled core orbital.^81^ Yet it yields mostly no covalent bonding contributions, although the Coulomb attraction to the neighbors must be substantial. In its way, this δ^4^ ring resembles the σ^2^ lone pairs. An open question is, what kind of chemistry could one do with the δ^4^?

## Footnotes

a The PW in the sequel refers to “present work” at this PBE0/TZ2P level.

b For brevity, we use here the atomic spectroscopy notation NQCC = *e*^2^*qQ*/*h* = *B*.
